# Regional Sub-Saharan Africa Total Diet Study in Benin, Cameroon, Mali and Nigeria Reveals the Presence of 164 Mycotoxins and Other Secondary Metabolites in Foods

**DOI:** 10.3390/toxins11010054

**Published:** 2019-01-17

**Authors:** Luc Ingenbleek, Michael Sulyok, Abimbola Adegboye, Sètondji Epiphane Hossou, Abdoulaye Zié Koné, Awoyinka Dada Oyedele, Chabi Sika K. J. Kisito, Yara Koreissi Dembélé, Sara Eyangoh, Philippe Verger, Jean-Charles Leblanc, Bruno Le Bizec, Rudolf Krska

**Affiliations:** 1 Centre Pasteur du Cameroun (CPC), Yaoundé BP1274, Cameroon; luc.ingenbleek@gmail.com (L.I.); eyangoh@pasteur-yaounde.org (S.E.); 2 LABERCA, Oniris, INRA, 44307 Nantes, France; Bruno.LeBizec@oniris-nantes.fr; 3 Department IFA-Tulln, University of Natural Resources and Life Sciences, Vienna (BOKU), 3430 Tulln, Austria; michael.sulyok@boku.ac.at (M.S.); Rudolf.Krska@boku.ac.at (R.K.); 4 National Agency for Food and Drug Administration and Control (NAFDAC), Abuja 900288, Nigeria; adegboye.a@nafdac.gov.ng (A.A.); oyedele.dada@nafdac.gov.ng (A.D.O.); 5 Agence Béninoise de Sécurité Sanitaire des Aliments (ABSSA), Cotonou BP 362, Benin; hossepfr@yahoo.fr; 6 Agence Nationale de la Sécurité Sanitaire des Aliments (ANSSA), Bamako BP 2362, Mali; ngolona@gmail.com; 7 Laboratoire Central de Sécurité Sanitaire des Aliments (LCSSA), Cotonou BP 6874, Benin; kinnousika@yahoo.fr; 8 Laboratoire de Technologie Alimentaire (LTA), Bamako BP 258, Mali; ykoreissidemb@gmail.com; 9 World Health Organization (WHO), 1211 Geneva, Switzerland; vergerp@who.int; 10 Food and Agriculture Organization of the United Nations (FAO), 00153 Rome, Italy; 11 Institute for Global Food Security, School of Biological Sciences, Queens University Belfast, Belfast BT7 1NN, Northern Ireland, UK

**Keywords:** Sub-Saharan Africa, aflatoxins, mycotoxins, total diet study, food contaminants, LC-MS/MS

## Abstract

In the framework of the first multi-centre Sub-Saharan Africa Total Diet Study (SSA-TDS), 2328 commonly consumed foods were purchased, prepared as consumed and pooled into 194 composite samples of cereals, tubers, legumes, vegetables, nuts and seeds, dairy, oils, beverages and miscellaneous. Those core foods were tested for mycotoxins and other fungal, bacterial and plant secondary metabolites by liquid chromatography, coupled with tandem mass spectrometry. The highest aflatoxin concentrations were quantified in peanuts, peanut oil and maize. The mean concentration of the sum of aflatoxins AFB1, AFB2, AFG1 and AFG2 (AF_tot_) in peanut samples (56.4 µg/kg) exceeded EU (4 µg/kg) and Codex (15 µg/kg) standards. The AF_tot_ concentration (max: 246.0 µg/kg) was associated with seasonal and geographic patterns and comprised, on average, 80% AFB1, the most potent aflatoxin. Although ochratoxin A concentrations rarely exceeded existing Codex standards, it was detected in unregulated foods. One palm oil composite sample contained 98 different metabolites, including 35.4 µg/kg of ochratoxin A. In total, 164 different metabolites were detected, with unspecific metabolites like asperglaucide, cyclo(L-pro-L-val), cyclo (L-pro-L-tyr), flavoglaucin, emodin and tryptophol occurring in more than 50% of composite samples. Aflatoxin B1 (AFB1), fumonisin B1 (FB1), sterigmatocystin (STC), ochratoxin A (OTA), citrinin (CIT) and many other secondary fungal metabolites are frequent co-contaminants in staple foods, such as maize and sorghum. Populations from North Cameroon and from Benin may, therefore, suffer chronic and simultaneous exposure to AFB1, FB1, STC, OTA and CIT, which are prevalent in their diet.

## 1. Introduction

Mycotoxins are secondary metabolites produced by filamentous fungi in food commodities due to inadequate pre- or post-harvest conditions and practices. These fungal toxins are, therefore, naturally-occurring chemical hazards. Since they are structurally stable, mycotoxins are likely to persist in foods, even if toxin-producing moulds are eliminated during the food preparation process. Consumption of mycotoxin-contaminated food may result in acute or chronic affections, including non-communicable diseases. A particularly severe record of acute toxicity was reported after a major outbreak struck Kenya in 2004, resulting in 317 aflatoxicosis cases including 125 deaths [1]. This episode was the consequence of high exposure to aflatoxins due to the consumption of extensively-contaminated maize [2]. Long-term exposure to aflatoxin B1 or its precursors has been associated with genotoxicity and hepatocellular carcinoma [3,4]. Fumonisin B1 was associated with oesophageal cancer incidence in South Africa and some areas of China [5,6]. Growth impairment, the main indicator for child chronic malnutrition, is also associated with mycotoxin exposure [7,8,9,10]. Of the world’s 161 million stunted children in 2013, about half live in Asia and over one-third live in Africa [11]. Although often overlooked as a possible cause of retarded growth, mycotoxins may contribute a significant public health burden in less developed countries [12].

An additive or synergistic effect of fumonisin and aflatoxin co-exposure in the development of preneoplastic lesions or hepatocellular carcinoma was suggested in laboratory animals [13,14,15].

Mycotoxins form the group of food chemicals which triggered the most cases of border rejection (489) recorded in the EU Rapid Alert System on Food and Feed [16]. According to the European Commission Regulation 1881/2006 [17], the maximum level for aflatoxins for peanuts and cereals intended for direct human consumption was set to 2 µg/kg of aflatoxin B1 (AFB1) and 4 µg/kg of the sum of AFB1, AFB2, AFG1 and AFG2. The maximum limit from the international standard [18] is 15 µg/kg of AFB1 or AF_tot_, which only applies to a variety of nuts (including peanuts) intended for further processing (and 10 µg/kg for ready to eat dried figs, almonds, hazelnuts and pistachios).

In order to assess if the chronic intake of substances is likely to harm consumer health, it is pertinent to assess food safety risks by combining available toxicological studies, as well as food contamination and food consumption data.

One way of assessing the dietary exposure of populations to food chemicals, such as mycotoxins is the Total Diet Study (TDS) approach [19,20,21,22,23,24]. Two specific aspects characterize a TDS—(1) the representativeness of the sampling, and (2) the preparation of the samples “as consumed”—so that it represents a pertinent public health risk assessment tool as far as food safety and nutrition are concerned.

The World Health Organization (WHO) and the Food and Agriculture Organization of the United Nations (FAO) endorse the TDS methodology, which is both cost-effective and more accurately characterizes human exposure to food chemicals than mere occurrence studies [23].

Following a first experience in Sub-Saharan Africa [25,26], a regional TDS was implemented by FAO in Benin, Cameroon, Mali and Nigeria (2014 and 2018) by four national food safety authorities, in close collaboration with WHO and Centre Pasteur of Cameroon [27]. The purpose of this project is to assess the typical contamination levels of eight African population groups. The dietary exposure of those population groups will then be compared with existing health-based guidance values or end points.

The study methodology was described elsewhere [28].

In this paper, we are presenting the occurrence of mycotoxins and selected fungal, bacterial and plant toxins quantified in composite samples of foods prepared as consumed. The 194 composites result from the pooling of 12 subsamples, representative of the food consumption habits of three study centres located in coastal areas (Duala, the Littoral of Benin and Lagos) and five study centres in non-coastal areas (Bamako, the Borgou region of Benin, Kano, North Cameroon and Sikasso).

## 2. Results

Since we are dealing with pooled samples (12 sub-samples per composite) of foods prepared as consumed in this study, we will not always be able to conclude with regard to the conformity of food commodities to selected standards [17,18], which, in most cases, apply to raw food commodities. This comparison is nonetheless useful, particularly when the mean concentration (quantified in a composite sample) exceeds or is close to the maximum legal limit of the substances of interest, because this means that at least one subsample out of 12 may have exceeded this limit.

Additionally, since these data will be used for a dietary exposure assessment, they are presented with (1) lower bound (i.e., LB: concentration of non-detected analytes set to zero and to the LOD for detected but non-quantified analytes) and (2) upper bound (i.e., UB: concentration of non-detected analytes set to LOD for non-detected analytes and to the limit of quantification (LOQ) for detected but non-quantified analytes) scenarios. This means that the uncertainty due to censored data will be taken into consideration. When LB–UB is not specified, it is meant that the difference between LB and UB concentrations in not perceptible or less than 0.1 µg/kg. Maximum concentration values are systematically UB concentrations.

Mycotoxins of public health and economic interest (including aflatoxins, fumonisins, ochratoxin A, zearalenone, deoxynivalenol and citrinin) represented 9% of the detected metabolites.

### 2.1. Aflatoxins

#### 2.1.1. Aflatoxins in Maize

Composite samples were prepared with maize from each study centre (8) purchased during the rainy season (October 2017) and again during the dry season or harmattan (February 2018).

The AF_tot_ concentration in maize was significantly higher (*p* < 0.05) during the wet season (detected: 7/8; mean LB–UB: 22.2–22.5 µg/kg; max: 76.6 µg/kg) than during the dry season (detected: 4/8; mean LB–UB: 0.4–0.8 µg/kg; max: 2.7 µg/kg). Overall, we detected that AF_tot_ > LOD in 11 of 16 composites (69%) with a mean LB–UB concentration of 11.3–11.7 µg/kg (Table 1) in ready-to-eat maize samples, which exceeds the EU standard for both processed (4 µg/kg) and unprocessed maize to be subjected to sorting or physical treatment before human consumption or use as an ingredient (10 µg/kg). However, the fact that all maize composites collected during the dry season contained AF_tot_ concentrations that were below 4 µg/kg and, therefore, complied with EU standard needs to be emphasized.

There is currently no Codex standard applicable to aflatoxins in maize. AFB1, the most potent aflatoxin, represented 87.6% of the sum of AFB1, AFB2, AFG1 and AFG2 detected in maize samples (Table 2).

#### 2.1.2. Aflatoxins in Peanut

As displayed in Table 1, the highest AF_tot_ concentration in this study was quantified in one peanut composite sample from Bamako (Mali): 246.0 µg/kg (mean LB–UB: 56.4–56.7 µg/kg). Aflatoxins were detected in 80% of peanut composites (rainy season: 100%, dry season: 60% detection exceeding LOD = 0.1 µg/kg). The mean AF_tot_ concentration in peanuts was 93.7–93.9 µg/kg (rainy season) and 19.1–19.4 µg/kg (dry season). A high variance of AF_tot_ levels in peanut was observed (CV > 100%). It was noted that while 50% of samples contained AF_tot_ concentrations below the EU standard (4 µg/kg) and 50% were above the Codex standard (15 µg/kg), the mean AF_tot_ concentrations exceeded both EU and Codex standards, regardless of the season. The proportion of AFB1 in peanut was 75.8% of the sum of AFB1, AFB2, AFG1 and AFG2 (Table 2).

#### 2.1.3. Aflatoxins in Peanut Oil

Two composite samples of peanut oil were tested (Table 1) and both contained significant amounts of total aflatoxins: 15.8 µg/kg (Kano) and 105.1 µg/kg (Cotonou). There is currently no standard for aflatoxins in oil, and these concentrations exceed Codex standards available for processed and unprocessed peanuts. The proportion of AFB1 in peanut oil was 86.6% of the sum of AFB1, AFB2, AFG1 and AFG2 (Table 2).

#### 2.1.4. Aflatoxins in Other Foods

Aflatoxins were detected in 60% of sorghum and 19% of bean composites. In Table 1, we reported that one bean sample contained 15.8 µg/kg AF_tot_. One smoked fish composite contained 4.9 µg/kg AF_tot_. The observed mean concentration of all tested core foods was below 1 µg/kg in Duala and Lagos (detection rate of 10%) but those recorded in North Cameroon exceeded 10 µg/kg (detection rate of 47%).

### 2.2. Fumonisins

FUM_tot_ (sum of fumonisins FB1, FB2, FB3 and FB4) were most concentrated in maize samples in all eight centres (Table 3). Although fumonisins were detected in 94% of ready-to-eat maize composites, all FUM_tot_ concentrations (mean LB–UB: 285.2–288.2 µg/kg; max: 855.9 µg/kg) remained below the Codex standard of 2 mg/kg applying to fumonisins in maize. Although there is no Codex standard for fumonisins in other foods than maize, other core food samples contained FUM_tot_ of up to 159.4 µg/kg. Apart from maize, composites containing fumonisins are sorghum (including a traditional fermented drink from North Cameroon processed from sorghum called bili-bili) and millet and tubers having undergone a drying process prior to being prepared as consumed (cassava and yam), as reported in Table 3. In food composites from Mali (Bamako and Sikasso), the mean FUM_tot_ concentration was three to ten-fold lower than in samples collected in the other study centres, with UB and LB scenario respectively. FUM_tot_ in our samples comprised 67.2% FB1, 18.9% FB2, 8.0% FB3 and 6.0% FB4. This is close to the proportions determined in maize samples (Table 4).

The co-occurrence of FB1 and AFB1 was observed in 11 of 16 maize composites (69%) and four of 10 sorghum composites (40%), as well as one of eight millet composites (13%) and in one of 12 cassava dry samples (8%).

### 2.3. Sterigmatocystin (STC)

STC, which is a known aflatoxin precursor [29] was mostly prevalent in cooking oils (Table 5). STC was quantified in 50% of peanut composites (mean: 0.6 µg/kg; max: 2.9 µg/kg) and in all peanut oil samples (mean: 8.5 µg/kg; max: 8.7 µg/kg), which also contained aflatoxins (Table 1). Interestingly, STC was quantified in 100% of “other vegetable oil” samples (cottonseed oil in most cases), whereas aflatoxins were not detected in those composites (tested with the same limit of detection, LD = 0.1 µg/kg).

Contrarily, STC detection rate in maize was only 13%, whereas aflatoxins were detected in 69% of composite samples.

There is currently no Codex or EU standard for STC in any food commodity.

The co-occurrence of STC, AFB1 and FB1 was observed in four composites samples, all collected during the rainy season:

1. Maize (North Cameroon): 56.6 µg/kg AFB1; 458.5 µg/kg FB1; 1.0 µg/kg STC
2. Maize (Benin Littoral): 71.8 µg/kg AFB1; 179.0 µg/kg FB1; 0.075 (LB = limit of detection)–0.25 µg/kg (UB = limit of quantification) of STC, which was detected below the limit of quantification.
3. Sorghum (Borgou): 1.7 µg/kg AFB1; 33.5 µg/kg FB1; 0.5 µg/kg STC
4. Sorghum (Sikasso): 0.8 µg/kg AFB1; 12.5 µg/kg FB1; 2.4 µg/kg STC

### 2.4. Ochratoxin A (OTA)

OTA was detected in 10% of tested composite samples (Table 6). Six percent (6%) of all tested samples exceeded 1 µg/kg OTA, including maize (13%), wheat (pasta 50%) and peanut oil (50%). Only three samples contained OTA concentrations exceeding Codex standards applying to unprocessed wheat, barley or rye (5 µg/kg): sorghum (Sikasso: 5.6 µg/kg), rice (Borgou: 6.3 µg/kg) and palm oil (Benin Littoral: 35.4 µg/kg).

There is currently no standard regulating OTA in edible oils, rice and sorghum.

### 2.5. Citrinin (CIT)

CIT was detected in 19% of all samples (Table 7), including maize (63%), sorghum (70%) and rice (38%). The only available citrinin standard (EU) applies to food supplements based on rice fermented by red yeast (2000 µg/kg). Ten percent (10%) of tested samples had CIT concentrations of 5 µg/kg or more and four maize composite samples exceeded 100 µg/kg (25% of maize samples and 2% of all samples).

### 2.6. Foods Contaminated by Other Regulated Mycotoxins

#### 2.6.1. Zearalenone (ZEN)

ZEN was detected in 6% of samples and never exceeded EU standards of 100 µg/kg for maize intended for direct human consumption. However, the three composite samples containing the highest ZEN concentrations were collected in the same study centre (Duala): maize (wet season: 7.6 µg/kg; dry season: 97.0 µg/kg) and cassava having undergone a drying process prior to being prepared as consumed (dry season: 7.6 µg/kg).

There is currently no Codex standard for ZEN in foods.

#### 2.6.2. Deoxynivalenol (DON)

DON was also detected in 6% of composite samples, including in (1) bread samples (detection rate: 100%) with a mean concentration of 68.8 µg/kg (min: 31.9 µg/kg; max: 134.6 µg/kg), (2) in 100% of pasta prepared as consumed (mean LB–UB: 9.8–14.3 µg/kg). This is inferior to Codex standards applying to DON cereal-based foods for children (200 µg/kg) and for wheat, maize and barley flour, meal, semolina and flakes (1000 µg/kg).

#### 2.6.3. Ergot Alkaloids

Twelve ergot alkaloids were detected in foods processed from wheat (5 of 6 bread samples), with a mean concentration 62.4 of µg/kg, ranging from non-detected to 165.7 µg/kg, for the sum of ergocornine (1.4%), ergocorninine (0.9%), ergocristine (21.0%), ergocristinine (6.8%), ergocryptine (7.6%), ergocryptinine (2.2%), ergometrine (14.4%), ergometrinine (0.5%), ergosin (21.0%), ergosinine (1.4%), ergotamin (21.7%) and ergotaminine (1.0%). There is no Codex standard for ergot alkaloids, and, to the best of our knowledge, the only available standard (EU) is 0.5 g/kg for the sum of ergot alkaloids in unprocessed cereals, except for maize and rice.

### 2.7. Non-Detected Mycotoxins of Health and Economic Significance

T2 and HT2 toxins, patulin and diacetoxyscirpenol were never detected in this present study.

### 2.8. Remarks on a Selection of Other Secondary Fungal, Bacterial and Plant Metabolites

#### 2.8.1. *Aspergillus fumigatus* Metabolites in Palm Oil

The presence of 11 *Aspergillus fumigatus* metabolites was observed in palm oil composites only. Bisdethiomethylgliotoxin was detected in three of four samples, with a mean (LB–UB) concentration of 117.7–118.0 µg/kg. Tryptoquivaline was detected in three of four samples (mean: 81.6–81.8 µg/kg). Gliotoxin was detected in three of four samples (mean: 36.6–36.2 µg/kg). Helvolvic acid was detected in two of four samples (mean 27.2–29.3 µg/kg). Fumigaclavin was detected in three of four samples (mean: 16.6–16.9 µg/kg). Fumagillin was detected in one of four samples (mean: 10.9–13.4 µg/kg). Methylsulochrin was detected in four of four samples (mean: 8.54 µg/kg). Pyripyropene A was detected in one of four samples (mean: 5.7–5.8 µg/kg). Fumitremorgin was detected in three of four samples (mean: 3.6–3.7 µg/kg). Pseurotin A was detected in two of four samples (mean: 2.1–2.8 µg/kg). Pyripyropene D was detected in one of four samples (mean 0.3–0.5 µg/kg). Little is known about these substances, which are not likely to represent a threat to consumer at these concentrations. Their presence, however, reveals that *Aspergillus fumigatus*, a human pathogen, may thrive in the palm oil production chain at some point between the palm tree and final production. Therefore, it represents a risk to value chain operators, if not to consumers [30].

#### 2.8.2. Cereulide in Smoked Fish

The bacterial metabolite cereulide was only detected five times (2.6%) in 194 samples, but was quantified in three of six or 50% of smoked fish samples. Mean (LB–UB) cereulide concentration in smoked fish was 0.8–0.9 µg/kg, and the maximum concentration was 2.5 µg/kg. The two other composites containing cereulide concentrations above the detection limit of 0.19 µg/kg were beef (2.0 µg/kg) and palm oil (0.7 µg/kg).

#### 2.8.3. Cyanogenic Glucosides in Cassava

Following TDS methodology, all samples were prepared as consumed, but a distinction was made between cassava samples having undergone size reduction, fermentation and drying processes (e.g., cossets or gari using dehydration as preservation and toxins reduction means) before preparation, including rehydration (cassava dry) [31], and other cassava samples (cassava fresh).

Exposure to cyanogenic glucosides, such as linamarin and lotaustralin, may cause serious motor neuron diseases, called konzo [32,33,34,35].

A seasonal pattern was observed, with higher concentrations of both linamarin and lotaustralin in fresh cassava during the dry season (*p* < 0.05), which was already reported by previous studies on the matter [36].

While linamarin concentrations ranged from below LD (2.3 µg/kg) to 317 mg/kg wet weight (mean: 134 mg/kg) in cassava fresh samples, it was quantified between 0.15 mg/kg and 18 mg/kg (mean: 2.8 mg/kg) in cassava dry composite samples (1:47 ratio).

Similarly, lotaustralin ranged from 0.04 mg/kg to 0.66 mg/kg (mean: 0.16 mg/kg) in cassava dry and from below LD (1.3 µg/kg) to 18 mg/kg (mean: 6.1 mg/kg) in cassava fresh (1:26 ratio).

Overall, linamarin and lotaustralin were less concentrated in dry cassava samples (*p* > 0.05) than in fresh cassava. The wide range of cyanogenic glucoside concentrations in dry cassava composites (max/min ratio of 120:1 in the case of linamarin and 510:1 for lotaustralin) may be explained by different processing practices, such as the use of the wetting method in cassava flour [37,38], although we were not able to verify these aspects from information requested during the collection of samples.

Four composite samples of cassava fresh were collected in each country during the wet season and again during the dry season or harmattan. Surprisingly, neither linamarin, nor lotaustralin were detected above LD (2.3 and 1.3 µg/kg wet weight, respectively) in samples collected in Nigeria, whereas concentrations varied from 93 to 101 mg/kg (wet season) and from 198 to 317 mg/kg (dry season) in Benin, Cameroon and Mali. We have not figured out the reason of this Nigeria-specific pattern, which may include different cassava varieties or cultivars [39] as well as different cooking methods [40,41,42].

#### 2.8.4. Low Contaminated Core Foods

We observed relatively low or no occurrence of mycotoxins and other toxins in foods prepared from fresh yam without dehydration processes, in rice and in traditional, soft and fermented drinks, as well as in sugar, onion, garlic, and eggs.

### 2.9. Secondary Metabolites Profile

**Figure 1:**
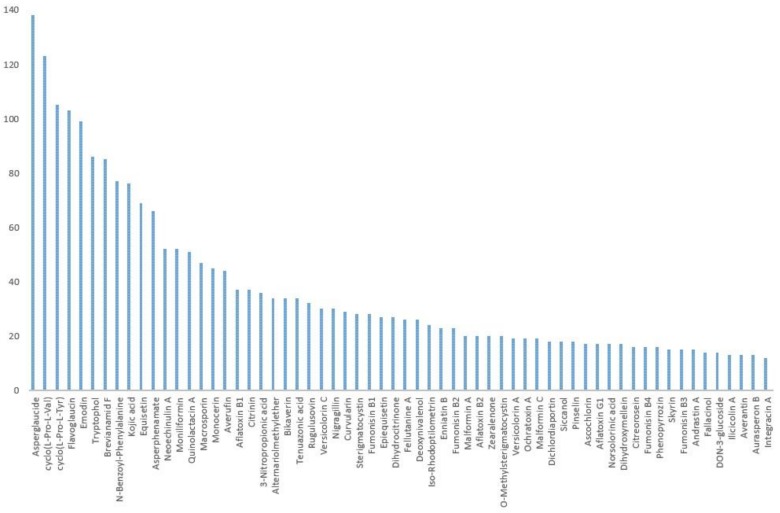
Most frequently detected secondary metabolites in Sub-Saharan Africa Total Diet Study (SSA-TDS) composite samples.

Figure 1 shows 62 of the most frequently occurring metabolites out of 164 analytes, on the basis of detection in our samples.

More than a third (36%) of detected metabolites are unspecific to any fungi genera and might also be of plant origin.

Among most prevalent metabolites, six were detected in more than 50% of samples:

• asperglaucide (141 samples or 73% of 194 samples);
• cyclo(L-pro-L-val) (138 samples or 71%);
• cyclo(L-pro-L-tyr) (123 samples or 63%);
• flavoglaucin (105 samples or 54%);
• emodin (103 samples or 53%); and
• tryptophol (99 samples or 51%).

Crude red palm oil composite samples from Cotonou (Benin), Lagos and Kano (Nigeria) showed a higher number of metabolite concentrations above LOD than any other sample (n: 3; mean: 88; min–max: 83–98 metabolites), as noted in Table 8.

All analytical data including quality control checks are enclosed in Table S1.

## 3. Discussion

First of all, the fact that 164 metabolites were detected in typical African foods does not mean that all of them represent a threat to human health. As of now, in the case of many analytes, the lack of knowledge on their toxicity and their combined (and potentially synergistic) effect with other substances limits our interpretation.

It does, however, represent a contribution to knowledge which may be used when new toxicological data with regards to some of these metabolites will be available. Therefore, the uniqueness of the multi-analyte LC–MS/MS approach used in this study, which enabled the occurrence characterization of a wide range of toxins and other fungal, plant and bacterial secondary metabolites, needs to be emphasized.

In the rest of this discussion, we found it relevant to focus on mycotoxins of public health and international trade significance.

The prevalence of mycotoxins in maize and peanut samples, though, has often been highlighted in previous surveys [43].

Worldwide, several total diet studies from various countries have included mycotoxins, including France [44,45,46], Canada [47], Lebanon [48], Vietnam [49], and China [50].

In Africa, studies of urinary biomarkers [51,52,53], surveys of food commodities [54,55], and the analysis foods prepared as consumed [56], have contributed to the rise in attention of the public health community to the threat that mycotoxins represent.

Unsurprisingly, the high concentration of the sum of AFB1, AFB2, AFG1 and AFG2 in African foods as consumed is probably the most significant public health and trade outcome of this multi-mycotoxin analysis compared with other regions of the world [57]. The fact that peanut oil may contain high AF_tot_ concentrations has only recently been described [58]. Peanut oil, peanut and maize are, therefore, likely to contribute significantly to AFB1 exposure, which will be used to characterize the risk of hepatocellular carcinoma.

The presence of fumonisins in staple foods such as maize, with concentrations below the Codex standard of 2 mg/kg, does not guarantee safety for our study populations. The Joint Expert Committee on Food Additives and Contaminants (JECFA) noted in the 83rd session [14], that the current worldwide exposure estimate was established with occurrence data belonging to countries of the WHO European region, and there was no available information on fumonisin levels in maize from the African, Eastern Mediterranean and South-East Asia regions. The JECFA also noted [14] that the interaction between AFB1, a compound with known genotoxic properties, and fumonisins, which have the potential to induce regenerative cell proliferation, is a concern. The completion of the dietary exposure assessment to the sum of FB1, FB2, FB3 and FB4 with the data presented in this paper will also result in conclusions with regards to the adequacy of protective levels of current Codex tolerances in the context of Africa (manuscript in preparation).

Surprisingly, the presence of a high concentration of AF precursor sterigmatocystin, not only in peanut oil but also in cottonseed oil and palm oil, was noted. In contrast, AF was never detected in cottonseed oil but detected in only one of four palm oil samples (0.5 µg/kg AFB1 in a red palm oil composite from sub-samples collected in the Littoral of Benin). This may be due to the production of STC by non-aflatoxigenic *Aspergilli*, such as *Aspergillus nidulans* [59], as well as other fungi genera [60]. The fact that we quantified STC in millet and sorghum composite samples is consistent with recent findings in sorghum [55]. Sorghum and millet, therefore, also qualify as potential STC dietary exposure contributors, noting that typical Sub-Saharan-Sahelian diets largely rely on these cereals [28].

The fact that citrinin was most concentrated in maize means that maize is likely to be a major contributor to CIT dietary exposures in centres where (1) maize CIT concentrations were high and (2) maize is consumed in large amounts.

We would like to bring forward the absence of Codex standards for mycotoxins in edible oils and, in light of occurrence data submitted in this paper, the need for surveillance of mycotoxin contamination levels in edible oils. The presence of (1) OTA in one palm oil with 97 other secondary metabolites, (2) high AF_tot_ concentrations in peanut oil, and (3) the presence of STC in cotton seed oil supports the need for an elaboration in the Codex code of practice for the production of safe edible oil.

Results of the risk characterization (manuscript in preparation) using this occurrence data and adequate food consumption data will clarify to what extent edible oils, as well as other core foods, contribute to the total dietary exposure to mycotoxins in Africa.

Mycotoxin exposure risk mitigation measures include growth prevention of toxin-producing fungi via biocontrol [61] in the field, good post-harvest practices [62] and mycotoxin degradation [63].

As human co-exposure to natural toxins through typical African foods is currently inevitable, national food safety authorities need to ensure that risk assessments are carried out properly to safeguard human health and to maintain international trade.

As demonstrated by the current study, AFB1, FB1, STC and many other secondary fungal metabolites are frequent co-contaminants in many foods (such as maize and sorghum) that threaten human health. Populations in North Cameroon and from Benin (where multiple toxins, including ABF1, FB1 and STC, have been detected within the TDS) may suffer repeated simultaneous exposure to natural toxins. In a recent study [15], the combined effects of various toxins at realistic concentrations were further investigated and revealed additive, antagonistic or synergistic effects. The results have confirmed that combinations of toxins may pose a considerable risk to human health. Clearly, further research is needed to understand the mechanics of toxicological interactions in order to effectively protect public health. Moreover, more TDSs in other locations of Benin, Cameroon, Mali and Nigeria, as well as in other countries belonging to Sub-Saharan Africa need to be carried out to better document actual dietary exposure levels to natural toxins in this region.

## 4. Conclusions

At this stage of the SSA-TDS project, the first ever multi-centric total diet study carried out in Africa, we have detected 164 secondary metabolites. However, our main results with regards to the occurrence of regulated mycotoxins in eight study centres are as follows:

• Mean AF_tot_ concentrations exceed EU and Codex tolerances applying to peanuts. Similar AF_tot_ levels were quantified in peanut oil (although no Codex or EU standards are currently available for edible oils), as well as in maize samples (aflatoxins in maize are not currently regulated by Codex).
• The TDS approach allowed for the capture of seasonal variations of the AF_tot_ contamination pattern in maize, which contains higher concentrations in samples collected during the rainy season.
• The geographic component of the AF_tot_ contamination pattern was suggested by variations in the mean AF_tot_ concentrations among study centres, which was also observed between two study centres from the same country (Duala versus North Cameroon).

Due to the systematic approach applied to this study, we consider these data fit for the completion of chronic dietary exposure assessment of mycotoxins, for which a health-based guidance value is available (e.g., Tolerable Daily Intake (TDI) or end point for genotoxic carcinogenic substances using the margin of exposure approach). We will then be able to take into consideration food consumption data, at the household level, for eight population groups. We expect maize, peanut and peanut oil to contribute to most of the dietary exposure to AFB1. Likewise, we expect maize to contribute highly to FUM_tot_ and CIT dietary exposure. However, other core foods, in which lower mycotoxin concentrations were estimated, especially highly-consumed staple foods, may also significantly contribute to households’ total dietary exposure.

Although Codex maximum limits were not exceeded in the case of FUM_tot_ and OTA, a household dietary exposure assessment will enable risk characterization of the investigated population groups. From this exercise, we will be able to conclude whether currently available Codex mycotoxin standards are sufficiently protective to African consumers.

The dietary exposure assessment of our study populations (manuscript in preparation) will provide guidance to risk managers from Benin, Cameroon, Mali and Nigeria for the identification of national priorities to the consumer protection agenda. We can nonetheless readily address our recommendations to risk managers based on AF_tot_ occurrence data referencing Codex standards only. It will indeed be beneficial for health and trade if national food safety authorities, with the support of their technical and financial partners, draft and implement a road-map and mobilize adequate resources taking the following into consideration:

• Food commodity value chain structures and organization;
• Prevention of field contamination by toxin-producing fungi; and
• Post-harvest practices with emphasis on hygiene, drying and storage conditions.

This will reduce the occurrence and concentrations of mycotoxins in African foods.

To date, these observations about STC occurrence in maize and in oils are new findings which were not reported or highlighted by the last JECFA evaluation of mycotoxins (2016) due to a lack of data at the time of the assessment.

Mitigation measures from Codex Alimentarius may include the updating of current codes of practices and standards and the elaboration of new ones to contribute to the reduction of natural toxins occurrence. This is in an effort to effectively safeguard African consumers’ health and food quality.

## 5. Experimental

### 5.1. Sample Selection and Preparation of Foods as Consumed

Food consumption data were derived from household budget surveys generated by National Statistics Authorities, from Benin, Cameroon, Mali and Nigeria and gathering a total of 72,979 households. Core foods of each study centre were selected based on the relative importance of their mean consumption [28], so as to cover at least 90% of the mean total diet in grams per adult male equivalent per day (g/AME/d).

Each core food was sampled through available representation criteria [64] (such as market share or the origins of the food) using 12 subsamples of equal size, prepared as consumed and pooled into composites, which underwent laboratory tests. The subsamples were prepared individually according to recipe books [65,66,67,68]. These references are considered as representative of the diet of the study populations and were, therefore, selected by the representatives of national competent authorities. These recipe books allow the identification of the processes used in the preparation of the foods, especially cooking time and temperature. The actual recipes were, however, not prepared as each composite sample only contained one core food or ingredient. The inedible parts were removed at the preparation stage, as a typical consumer would do. Distilled water instead off tap water was used to prepare food as consumed to avoid contamination. The quantity of water added during the cooking process of each of the 12 subsamples was measured by weighing the food at each stage of the preparation process.

Two seasons were captured [69] for five main food groups, which cover staple foods and most of the mean total diet by weight (i.e., cereals, tubers, legumes, vegetables and fruits):

• The rainy season in October 2017; and
• The dry season, or harmattan, in February 2018.

Other food groups were collected during the rainy season only (i.e., nuts and seeds, dairy, oils, beverages and miscellaneous).

Among 335 composite samples, 194 consisted of foods which may be stored in conditions allowing for the growth of moulds and, consequently, are likely to comprise mycotoxins. Those 194 composite samples were selected for mycotoxin analysis. Samples were frozen and shipped by air in coolers with dry ice, within a timeframe never exceeding 24 hours, from the kitchen laboratory (Benin, Cameroon, Mali and Nigeria) to the testing laboratory (Austria).

### 5.2. Reagents and Chemicals

LC gradient grade methanol and acetonitrile, as well as MS grade ammonium acetate and glacial acetic acid (p.a.), were purchased from Sigma Aldrich (Vienna, Austria). A Purelab Ultra system (ELGA LabWater, Celle, Germany) was used for further purification of reverse osmosis water.

Standards of fungal and bacterial metabolites were obtained either as gifts from various research groups or from the following commercial sources: Romer Labs^®^Inc. (Tulln, Austria), Sigma-Aldrich (Vienna, Austria), BioAustralis (Smithfiled, Australia), AnalytiCon Discovery (Potsdam, Germany), Fermentek (Jerusalem, Israel), Iris Biotech GmbH (Marktredwitz, Germany), Enzo Life Sciences Europe (Lausanne, Switzerland) and LGC Promochem GmbH (Wesel, Germany). Stock solutions of each analyte were prepared by dissolving the solid substance in acetonitrile, acetonitrile/water 1:1 (*v*/*v*), methanol, methanol/water 1:1 (*v*/*v*) or water. Thirty-four combined working solutions were prepared by mixing the stock solutions of the corresponding analytes for easier handling and were stored at −20 °C. The final working solution was freshly prepared prior to spiking experiments through mixing of the combined working solutions.

### 5.3. Laboratory Sample Preparation

Twenty millilitres (20 mL) of extraction solvent (acetonitrile/water/acetic acid 79:20:1, *v*/*v*/*v*) were added to 5 g of sample. The samples were extracted for 90 minutes using a GFL 3017 rotary shaker (GFL, Burgwedel, Germany) and subsequently centrifuged for two minutes at 3000 rpm (radius 15 cm) on a GS-6 centrifuge (Beckman Coulter Inc., Fullerton, CA, USA). The extracts were diluted (1:1) with dilution solvent (acetonitrile/water/acetic acid 20:79:1, *v*/*v*/*v*). After appropriate mixing, 5 μL of the diluted extract was injected into the LC-MS/MS system without further pre-treatment.

### 5.4. LC-MS/MS Parameters

Metabolite analysis was carried out using a 1290 Series HPLC System (Agilent, Waldbronn, Germany) coupled to a QTrap 5500 LC-MS/MS System (Applied Biosystems SCIEX, Foster City, CA, USA) equipped with Turbo Ion Spray electrospray ionization source, as described earlier [70]. Chromatographic separation was performed at 25 °C on a Gemini^®^ C_18_-column (150 × 4.6 mm i.d., 5 μm particle size) equipped with a C_18_ 4 × 3 mm i.d. security guard cartridge (Phenomenex, Torrance, CA, USA). Confirmation of positive metabolite identification was carried out by two instances of scheduled multiple reaction monitoring (MRMs) which yielded 4.0 identification points according to the European Commission decision 2002/657 [71].

In order to further decrease the limits of detection (LODs) for aflatoxin B1 and ochratoxin A, larger aliquots of 20 µL of the diluted extracts (previously fortified with the related ^13^C-labelled internal standards) were re-analysed using the QTrap 6500 LC-MS/MS system while keeping all other method parameters constant.

### 5.5. Quantification and Quality Control

Quantification was performed using external calibration based on serial dilution of a multi-analyte stock solution. Results were corrected using apparent recoveries that were determined for each of the investigated matrices by spiking experiments. The accuracy of the method is verified on a continuous basis by participation in a proficiency testing scheme organized by BIPEA (Gennevilliers, France) with a current success rate (i.e., a z-score between −2 and 2) of >94% of the >900 results submitted.

## Figures and Tables

**Table 1:** Occurrence and concentration of total aflatoxins (µg/kg wet weight) by core food and by study centre.

CORE FOOD		N	n > LOD	% > LOD	n > 4 µg/kg	% > 4 µg/kg	n > 15 µg/kg	% > 15 µg/kg	Mean Conc. *		Max Conc.	
									LB	UB	Season **	UB
**Maize**		16	11	69	5	31	3	19	11.3	11.7	Rainy	76.6
**Peanut**		10	8	80	5	50	5	50	56.4	56.7	Rainy	246.0
**Peanut oil**		2	2	100	2	100	2	100	60.2	60.4	Rainy	105.1
**Beans**		16	3	19	1	6	1	6	1.2	1.6	Dry	15.8
**Sorghum**		10	6	60	1	10	0	0	0.9	1.3	Rainy	4.9
**Smoked fish**		6	1	17	1	17	0	0	0.8	1.1	Rainy	4.9
**Other core foods**		134	11	8	0	0	0	0	0.1	0.5	Rainy	2.4
**Total**		194	42	22	15	8	11	6	4.7	5.1	Rainy	246.0
**CENTRE**		**N**	**n > LD**	**% > LD**	**n > 4 µg/kg**	**% > 4 µg/kg**	**n > 15 µg/kg**	**%> 15 µg/kg**	Mean Conc. *		**Max Conc.**	
									**LB**	**UB**	**Core food**	**UB**
**BENIN**	**Littoral**	26	5	19	3	12	3	12	7.6	8.0	Peanut oil	105.1
	**Borgou**	22	7	32	1	5	1	5	1.2	1.6	Maize	19.7
**CAMEROON**	**Duala**	29	3	10	0	0	0	0	0.2	0.6	Beans	3.0
	**North**	17	8	47	4	24	3	18	14.3	14.6	Peanuts	92.5
**MALI**	**Bamako**	27	4	15	2	7	1	4	9.4	9.8	Peanuts	246.0
	**Sikasso**	21	6	29	1	5	1	5	2.2	2.6	Peanuts	42.7
**NIGERIA**	**Lagos**	29	3	10	1	3	0	0	0.2	0.6	Maize	5.4
	**Kano**	23	6	26	3	13	3	13	5.6	6.0	Peanuts	96.6

* LB: lower-bound scenario where the concentration of non-detected analyte is zero and the concentration of detected but non-quantified analyte is the limit of detection. UB: upper-bound scenario where the concentration of non-detected analyte is the limit of detection and the concentration of detected but non-quantified analyte is the limit of quantification; ** Samples of the rainy season were collected in October 2017 and samples of the dry season were collected in February 2018.

**Table 2:** Proportions of aflatoxin B1, B2, G1 and G2 by core food and by weight.

CORE FOOD	AFB1 (%)	AFB2 (%)	AFG1 (%)	AFG2 (%)	Sum (%)
**Maize**	87.6	6.8	5.6	0.0	100
**Peanut**	75.8	14.3	9.4	0.5	100
**Peanut oil**	86.6	13.1	0.3	0.0	100
**Other core foods**	87.0	4.0	9.1	0.0	100
**Total**	80.1	12.1	7.5	0.3	100

**Table 3:** Occurrence and concentration of total fumonisins (µg/kg wet weight) by core food and by study centre.

CORE FOOD		N	n > LOD	% > LOD	n > 10 µg/kg	% > 10 µg/kg	n > 400 µg/kg	% > 400 µg/kg	Mean Conc. *		Max Conc.	
									LB	UB	Season **	UB
**Maize**		16	15	94	15	94	4	25	285.2	288.2	Dry	855.9
**Sorghum**		10	5	50	5	50	0	0	20.0	36.1	Dry	159.4
**Millet**		8	1	13	1	13	0	0	5.0	13.6	Rainy	44.8
**Traditional fermented drink**		4	1	25	1	25	0	0	5.7	14.1	Rainy	29.3
**Cassava dry**		12	3	25	3	25	0	0	14.8	22.9	Dry	134.6
**Yam dry**		2	1	50	1	50	0	0	7.4	17.8	Rainy	21.7
**Other core foods**		142	2	1	0	0	0	0	0.04	9.2	Both	14.6
**Total**		194	28	14	26	13	4	2	26.4	34.8	Dry	855.9
**CENTRE**		**N**	**n > LOD**	**% > LOD**	**n > 10 µg/kg**	**% > 10 µg/kg**	**n > 400 µg/kg**	**% > 400 µg/kg**	Mean Conc. *		**Max Conc.**	
									**LB**	**UB**	**Core food**	**UB**
**BENIN**	**Littoral**	26	2	8	2	8	0	0	26.8	35.2	Maize	391.3
	**Borgou**	22	5	23	5	23	0	0	26.3	34.4	Maize	376.5
**CAMEROON**	**Duala**	29	5	17	4	14	0	0	19.0	27.1	Maize	241.7
	**North**	17	12	71	5	29	1	6	64.4	71.6	Maize	670.3
**MALI**	**Bamako**	27	3	11	2	7	0	0	2.0	11.0	Maize	40.6
	**Sikasso**	21	2	10	2	10	0	0	4.1	12.9	Maize	79.0
**NIGERIA**	**Lagos**	29	4	14	3	10	1	3	34.9	43.5	Maize	855.9
	**Kano**	23	3	13	3	13	2	9	45.9	54.1	Maize	589.9

* LB: lower-bound scenario where the concentration of non-detected analyte is zero and the concentration of detected but non-quantified analyte is the limit of detection. UB: upper-bound scenario where the concentration of non-detected analyte is the limit of detection and the concentration of detected but non-quantified analyte is the limit of quantification; ** Samples of the rainy season were collected in October 2017 and samples of the dry season were collected in February 2018.

**Table 4:** Proportions of fumonisins B1, B2, B3 and B4 by core food and by weight.

CORE FOOD	FB1 (%)	FB2 (%)	FB3 (%)	FB4 (%)	Sum (%)
**Maize**	65.9	19.3	8.4	6.4	100
**Sorghum**	76.7	15.8	4.6	2.8	100
**Cassava dry**	75.4	14.1	6.2	4.2	100
**Other core foods**	88.2	11.8	0.0	0.0	100
**Total**	67.2	18.9	8.0	6.0	100

**Table 5:** Occurrence and concentration of sterigmatocystin (µg/kg wet weight) by core food and study centre.

CORE FOOD		N	n > LOD	% > LOD	n > 1 µg/kg	% > 1 µg/kg	n > 4 µg/kg	% > 4 µg/kg	Mean Conc. *		Max Conc.	
									LB	UB	Season **	UB
**Peanut oil**		2	2	100	2	100	2	100	8.5	8.5	Rainy	8.7
**Peanut**		10	5	50	2	20	0	0	0.6	0.6	Rainy	2.9
**Palm oil**		4	3	75	3	75	1	25	2.0	2.0	Rainy	5.3
**Other vegetable oil**		4	4	100	3	75	1	25	3.9	3.9	Rainy	9.2
**Sorghum**		10	3	30	2	20	0	0	0.4	0.5	Rainy	2.4
**Millet**		8	2	25	1	13	1	13	0.6	0.7	Rainy	4.8
**Other core foods**		156	10	6	0	0	0	0	0.02	0.1	Rainy	1.0
**Total**		194	29	15	13	7	5	3	0.3	0.4	Rainy	9.2
**CENTRE**		**N**	**n > LOD**	**% > LOD**	**n > 1 µg/kg**	**% > 1 µg/kg**	**n > 4 µg/kg**	**% > 4 µg/kg**	Mean Conc. *		**Max Conc.**	
									**LB**	**UB**	**Core food**	**UB**
**BENIN**	**Littoral**	26	4	15	2	8	1	4	0.4	0.5	Peanut oil	8.3
	**Borgou**	22	2	9	0	0	0	0	0.03	0.1	Sorghum	0.5
**CAMEROON**	**Duala**	29	2	7	1	3	0	0	0.1	0.2	Other vegetable oil	3.0
	**North**	17	4	24	1	6	1	6	0.7	0.7	Other vegetable oil	9.2
**MALI**	**Bamako**	27	4	15	3	11	1	4	0.3	0.4	Millet	4.8
	**Sikasso**	21	6	29	3	14	0	0	0.4	0.4	Peanuts	2.9
**NIGERIA**	**Lagos**	29	3	10	1	3	1	3	0.2	0.3	Palm oil	5.3
	**Kano**	23	4	17	2	9	1	4	0.5	0.6	Peanut oil	8.7

* LB: lower-bound scenario where the concentration of non-detected analyte is zero and the concentration of detected but non-quantified analyte is the limit of detection. UB: upper-bound scenario where the concentration of non-detected analyte is the limit of detection and the concentration of detected but non-quantified analyte is the limit of quantification; ** Samples of the rainy season were collected in October 2017 and samples of the dry season were collected in February 2018.

**Table 6:** Occurrence and concentration of ochratoxin A (µg/kg wet weight) by core food and by study centre.

CORE FOOD		N	n > LOD	% > LOD	n > 1µg/kg	% > 1µg/kg	n > 5µg/kg	% > 5µg/kg	Mean Conc. *		Max Conc.	
									LB	UB	Season **	UB
**Palm oil**		4	1	25	1	25	1	25	8.9	8.9	Rainy	35.4
**Rice**		16	5	31	4	25	1	6	0.9	0.9	Dry	6.3
**Sorghum**		10	2	20	2	20	1	10	0.8	0.9	Rainy	5.6
**Maize**		16	2	13	2	13	0	0	0.2	0.2	Rainy	1.4
**Peanut oil**		2	1	50	1	50	0	0	1.2	1.3	Rainy	2.5
**Pasta**		2	1	50	1	50	0	0	0.5	0.6	Rainy	1.1
**Other core foods**		144	7	5	0	0	0	0	0.03	0.1	Rainy	0.8
**TOTAL**		194	19	10	11	6	3	2	0.4	0.4	-	35.4
**CENTRE**		**N**	**n > LOD**	**% > LOD**	**n > 1 µg/kg**	**% > 1 µg/kg**	**n > 5 µg/kg**	**% > 5 µg/kg**	Mean Conc. *		**Max Conc.**	
									**LB**	**UB**	**Core food**	**UB**
**BENIN**	**Littoral**	26	5	19	5	19	1	4	1.6	1.6	Palm oil	35.4
	**Borgou**	22	3	14	3	14	1	5	0.4	0.5	Rice	6.3
**CAMEROON**	**Duala**	29	3	10	3	10	0	0	0.04	0.1	Cassava fresh	0.7
	**North**	17	3	18	3	18	0	0	0.2	0.3	Rice	2.0
**MALI**	**Bamako**	27	1	4	1	4	0	0	0.1	0.2	Maize	1.4
	**Sikasso**	21	1	5	1	5	1	5	0.3	0.4	Sorghum	5.6
**NIGERIA**	**Lagos**	29	0	0	0	0	0	0	0	0.1	ND	0.1
	**Kano**	23	2	9	3	13	0	0	0.2	0.3	Rice	2.6

* LB: lower-bound scenario where the concentration of non-detected analyte is zero and the concentration of detected but non-quantified analyte is the limit of detection. UB: upper-bound scenario where the concentration of non-detected analyte is the limit of detection and the concentration of detected but non-quantified analyte is the limit of quantification; ** Samples of the rainy season were collected in October 2017 and samples of the dry season were collected in February 2018.

**Table 7:** Occurrence and concentration of total citrinin (µg/kg wet weight) by core food and by study centre.

CORE FOOD		N	n > LOD	% > LOD	n > 5 µg/kg	% > 5 µg/kg	n > 100 µg/kg	% > 100 µg/kg	Mean Conc. *		Max Conc.	
									LB	UB	Season **	UB
**Maize**		16	10	63	9	56	4	25	76.4	76.8	Rainy	416.5
**Sorghum**		10	7	70	4	40	0	0	5.5	6.3	Rainy	18.2
**Rice**		16	6	38	3	19	0	0	2.8	3.2	Dry	18.0
**Other core foods**		152	14	9	4	3	0	0	0.3	1.0	Rainy	7.4
**TOTAL**		194	37	19	20	10	4	2	7.0	7.7	Rainy	416.5
**CENTRE**		**N**	**n > LOD**	**% > LOD**	**n > 5 µg/kg**	**% > 5 µg/kg**	**n > 100 µg/kg**	**% > 100 µg/kg**	Mean Conc. *		**Max Conc.**	
									**LB**	**UB**	**Core food**	**UB**
**BENIN**	**Littoral**	26	4	15	3	12	2	8	19.0	19.7	Maize	372.3
	**Borgou**	22	9	41	7	32	1	5	21.9	22.5	Maize	416.5
**CAMEROON**	**Duala**	29	4	14	2	7	1	3	5.7	6.5	Maize	123.6
	**North**	17	4	24	1	6	0	0	2.2	3.0	Maize	31.9
**MALI**	**Bamako**	27	2	7	0	0	0	0	0.1	0.9	Maize/Sorghum	2.5
	**Sikasso**	21	4	19	2	10	0	0	1.5	2.1	Sorghum	17.0
**NIGERIA**	**Lagos**	29	6	21	3	10	0	0	2.6	3.2	Maize	55.9
	**Kano**	23	4	17	2	9	0	0	3.4	4.0	Maize	61.4

* LB: lower-bound scenario where the concentration of non-detected analyte is zero and the concentration of detected but non-quantified analyte is the limit of detection. UB: upper-bound scenario where the concentration of non-detected analyte is the limit of detection and the concentration of detected but non-quantified analyte is the limit of quantification; ** Samples of the rainy season were collected in October 2017 and samples of the dry season were collected in February 2018.

**Table 8:** Range of metabolites detected by core food composite samples.

Range Number of Analytes (min-max)	Composite Samples (n)	CORE FOODS
**(51–98)**	3	Palm oil
**(21–50)**	46	Maize, dried tubers, sorghum, peanuts, bread, various oils
**(11–20)**	62	Beans, dried cassava, rice, millet, smoked fish, onion and garlic, fermented drinks
**(6–10)**	45	Onion and garlic, meat, tubers, dairy products, rice, traditional soft drinks
**(1–5)**	36	Fresh tubers, sugar, onion and garlic, rice, eggs
**0**	2	Onion and garlic
**(0–98)**	194	TOTAL

## Supplementary Materials

The following are available online at http://www.mdpi.com/2072-6651/11/1/54/s1. Table S1: Raw Analytical Data.
